# FSCA-EUNet: Lightweight Classification of Stacked Jasmine Bloom-Stages via Frequency–Spatial Cross-Attention for Industrial Scenting Automation

**DOI:** 10.3390/foods14213780

**Published:** 2025-11-04

**Authors:** Zhiwei Chen, Zhengrui Tian, Haowen Zhang, Xingmin Zhang, Xuesong Zhu, Chunwang Dong

**Affiliations:** Tea Research Institute, Shandong Academy of Agricultural Sciences, Jinan 250100, China; zv.chen@foxmail.com (Z.C.); tain981128@163.com (Z.T.); 18853875771@163.com (H.Z.); zhangxingmin9602@163.com (X.Z.); 15831111103@163.com (X.Z.)

**Keywords:** fine-grained classification, frequency–spatial cross-attention, postharvest jasmine, center loss, plant phenotyping

## Abstract

To address the challenge of monitoring the postharvest jasmine bloom stages during industrial tea scenting processes, this study proposes an efficient U-shaped Network (U-Net) model with frequency–spatial cross-attention (FSCA-EUNet) to resolve critical bottlenecks, including repetitive backgrounds and small interclass differences, caused by stacked jasmine flowers during factory production. High-resolution images of stacked jasmine flowers were first preprocessed and input into FSCA-EUNet, where the encoder extracted multi-scale spatial features and the FSCA module incorporated frequency-domain textures. The decoder then fused and refined these features, and the final classification layer output the predicted bloom stage for each image. The proposed model was designed as a “U-Net”-like structure to preserve multiscale details and employed a frequency–spatial cross-attention module to extract high-frequency texture features via a discrete cosine transform. Long-range dependencies were established by NonLocalBlook, located after the encoders in the model. Finally, a momentum-updated center loss function was introduced to constrain the feature space distribution and enhance intraclass compactness. According to the experimental results, the proposed model achieved the best metrics, including 95.52% precision, 95.42% recall, 95.40% F1-score, and 97.24% mean average precision, on our constructed dataset with only 878.851 K parameters and 15.445 G Floating Point Operations (FLOPs), and enabled real-time deployment at 22.33 FPS on Jetson Orin NX edge devices. The ablation experiments validated the improvements contributed by each module, which significantly improved the fine-grained classification capability of the proposed network. In conclusion, FSCA-EUNet effectively addresses the challenges of stacked flower backgrounds and subtle interclass differences, offering a lightweight yet accurate framework that enables real-time deployment for industrial jasmine tea scenting automation.

## 1. Introduction

Jasmine tea is a type of scented tea that is produced by reprocessing tea via the absorption of fresh jasmine flower fragrance. Jasmine tea has gained popularity because of its distinctive aroma and diverse health benefits, including antioxidant [1] and sedative properties [2]. The jasmine tea industry has demonstrated strong growth potential driven by product diversification, premiumization trends, and expanding consumer demand.

In industrial production, the scenting process critically depends on accurate monitoring of jasmine bloom stages, as fragrance absorption reaches its peak when flowers are at approximately 90% bloom [3]. Peak aroma development in postharvest jasmine buds is achieved through thermal stacking, which accelerates natural blooming via controlled heat accumulation. Conventional methods require laborious hourly pile turning for 2–3 h to prevent decay, and bloom-stage identification relies heavily on subjective human judgment. However, even small deviations in determining the optimal bloom time can directly affect tea aroma quality, product consistency, and economic value. Automated processing therefore requires precise and objective bloom-stage recognition, which is a critical yet challenging task due to the continuous blooming process, high visual similarity between stages, and small interclass variations.

The classification of flowers has a long history. With the advancement of computer vision, machine learning and deep learning have been extensively applied to flower classification [4]. Several publicly available datasets, such as Oxford-17 [5] and Oxford-102 [6], have gained extensive adoption in this field. Early studies primarily relied on manually designed features for flower recognition [7], which exhibited poor generalizability and low accuracy rates. In recent years, significant progress has been made in flower classification via deep learning. For example, advanced networks such as VGG [8] and Xception [9] have been employed for classification tasks [10,11,12], and they have significantly improved classification performance.

For the fine-grained recognition of different flower varieties within the same species, Yuan et al. [13] implemented a chrysanthemum variety classification system using an end-to-end convolutional neural network (CNN) to achieve fine-grained differentiation between chrysanthemum varieties. Several studies have investigated the detection of flowering bloom stages. Khanal et al. [14] proposed a YOLOv5-based visual system for detecting early-stage apple blossoms to guide flower thinning and pollination. Mu et al. [15] developed a Mask R-CNN-based detection model for identifying the blooming stages of apple flowers for robotic pollination. Li et al. [16] used YOLOv3 for kiwiflower and bud detection to estimate flowering peaks and enable robotic pollination. Notably, Pan et al. [17] introduced KIWI-YOLO, which integrated frequency-domain feature fusion to detect kiwiflower pollination, achieving 91.6% mean average precision (mAP) with only 1.8 M parameters. For quality classification of postharvest flowers, Sun et al. [18] tested four CNN models using four-dimensional RGBD inputs to improve classification performance. Fei et al. [19] established an architecture based on the ShuffleNet V2 and performed transfer learning to classify five specifications of fresh flowers, achieving classification accuracy exceeding 98%. These studies provided references for the methodology of this research. Recent advances in attention mechanisms have shown clear benefits for fine-grained recognition. For instance, CrossATF introduced cross-attention interaction for multi-modal fusion [20], BCAR-Net enhanced spatial representation through bidirectional cross-attention and attention restoration [21], and DLNet combined self- and cross-attention for high-resolution segmentation with state-of-the-art accuracy [22]. These studies demonstrate the strong potential of cross-attention, but also reveal challenges such as high computational cost or limited task adaptability. Motivated by their strengths, this study proposes a lightweight frequency–spatial cross-attention (FSCA) module to achieve both discriminative power and efficiency for postharvest jasmine flower classification.

Research on jasmine recognition and detection remains relatively limited, with a primary focus on field environments. Abinaya and Roomi [23] developed a superpixel-based segmentation algorithm that uses linear iterative clustering to generate superpixels, with DBSCAN clustering applied to flower cluster detection. Qur’ania and Sarinah [24] employed Sobel edge detection and k-nearest neighbor algorithms to identify different jasmine varieties. Amer et al. [25] designed a jasmine flower automatic picking system that integrated an Intel depth camera and YOLOv5 deep learning network for flower localization and detection, achieving average precision and recall rates of 100% and 90%, respectively. Zhou et al. [26] proposed a YOLOv7-based network for detecting jasmine flowers at various bloom stages in the field, attaining an mAP of 0.948. More recently, complementary jasmine-related research has also emerged in quality assessment and phenotyping. For instance, Zhu et al. [27] developed a computer vision and color difference approach for jasmine tea quality assessment, while Wei et al. [28] applied 3D point cloud analysis for jasmine plant phenotyping. These studies underscore the growing importance of jasmine and tea-related intelligent processing, further validating the practical value of our work.

Current research on jasmine flower detection and classification primarily focuses on field scenarios, aiming to detect different varieties or flowering stages to support precision agriculture needs. However, no studies have investigated classification methods for postharvest jasmine flowers in factory environments. Compared to field environments, factories exhibit significant differences in illumination conditions and operational environments. Furthermore, postharvest jasmine flowers in factories typically appear in stacked arrangements, resulting in highly repetitive image backgrounds and highly similar flower states, substantially increasing the difficulty of recognition and classification.

To address the challenges of repetitive backgrounds and interclass similarity in postharvest jasmine flower classification under the stacking status, this study proposed FSCA-EUNet, an efficient U-Net model with frequency–spatial cross-attention. The U-Net encoder–decoder design [29] has been successfully applied in various computer vision tasks. This research focused on leveraging its ability to capture both global context and local details for fine-grained classification. The proposed framework employs MBConv [30] in an efficient U-Net-like encoder–decoder architecture (efficient U-Net) and introduces a frequency–spatial cross-attention (FSCA) mechanism, NonLocalBlock, and momentum-updated center loss to improve fine-grained classification performance. The primary contributions of this study are as follows:

(1) The adoption of an MBConv-based efficient U-Net architecture with skip connections and transposed convolutions enables multiscale feature fusion, integrating high-level semantics with local detail localization, and mitigating spatial information loss caused by excessive downsampling;
(2) A FSCA mechanism is proposed, integrating DCT-derived high-frequency textures with spatial features via adaptive fusion and cross-channel attention to enhance texture discrimination under the stacking status;
(3) NonLocalBlock is introduced for global-local joint feature modeling, capturing long-range dependencies in floral morphology and fine-grained textural details while strengthening the global perception capacity of the network;
(4) A momentum-updated center loss function is designed to enhance the intraclass compactness and interclass separation of postharvest jasmine flower features through dynamic class center updates via a momentum mechanism and contrastive constraints, thereby improving fine-grained classification performance.

## 2. Related Work and Limitations

In recent years, encoder–decoder architectures have been widely adopted in computer vision tasks, particularly for image segmentation and classification. Among them, the U-Net architecture is one of the most representative designs, originally developed for biomedical image segmentation. Its success stems from the symmetric encoder–decoder structure, which enables the model to capture both high-level semantic representations and low-level spatial details through skip connections. However, despite its effectiveness, U-Net faces several challenges when applied to complex scenarios such as fine-grained classification or classification under highly repetitive backgrounds. A major limitation lies in its restricted ability to capture global context, which is essential for distinguishing subtle inter-class variations when visual similarities or occlusions occur.

Other variants of encoder–decoder architectures, such as SegNet [31] and DeepLab [32], have been proposed to alleviate these limitations. SegNet introduces a novel upsampling approach based on max-pooling indices, enhancing spatial detail recovery. Nevertheless, SegNet still struggles to discriminate small visual differences between classes with high inter-class similarity. In addition, both SegNet and U-Net mainly focus on spatial-domain features while neglecting frequency-domain cues that are critical for capturing fine textures and detailed structures. This limitation is particularly evident in fine-grained tasks where high-frequency information, such as edges and micro-textures, plays a vital role in classification accuracy.

The DeepLab series further advances this architecture by employing atrous convolution to preserve spatial resolution while capturing multi-scale contextual information. DeepLabv3 [33] enhanced this strategy using the atrous spatial pyramid pooling (ASPP) module, and DeepLabv3+ [34] introduced an encoder–decoder refinement process to better recover fine object boundaries. While these architectures achieved excellent accuracy, their computational demands remain high, limiting their suitability for real-time deployment on resource-constrained hardware.

To address these challenges, recent studies have incorporated attention mechanisms, such as self-attention and multi-head attention [35]. Attention-based models, including Attention U-Net and Transformer-based networks such as TransUNet [36] and DA-TransUNet [37], have demonstrated improved segmentation and classification performance by dynamically focusing on relevant spatial regions. These mechanisms strengthen global context modeling and long-range dependency capture, which is particularly beneficial for visually complex or fine-grained tasks. However, these models typically incur substantial computational costs, reducing their practicality for real-time or embedded applications.

Beyond model-level architectures, numerous feature enhancement modules have been proposed to improve representational efficiency. The Squeeze-and-Excitation (SE) module [38] adaptively recalibrates channel responses via global context aggregation, while the Convolutional Block Attention Module (CBAM) [39] extends attention to both channel and spatial dimensions. The more compact designs, such as Efficient Channel Attention (ECA) [40] and Coordinate Attention [41], achieve improved efficiency with lower computational overhead.

In addition to these spatial-domain attention mechanisms, frequency-domain attention has recently gained growing attention for its ability to capture global statistics and texture structures. FcaNet [42] pioneered this direction by introducing a multi-spectral channel attention mechanism based on the 2D discrete cosine transform (DCT), allowing the network to leverage frequency information for richer feature representation. Subsequently, FicNet [43] proposed multi-frequency neighborhood (MFN) and double-cross modulation (DCM) modules to jointly model frequency–spatial dependencies, thereby reducing intra-class variations and improving inter-class discrimination in few-shot fine-grained tasks. Similarly, FTransDF-Net [44] incorporated a lightweight frequency transformer and frequency attention module based on Fourier transform to capture long-range dependencies and enhance discriminative features in remote sensing image change detection. These studies collectively demonstrate the effectiveness of integrating frequency-domain cues into attention mechanisms for enhancing fine-grained feature discrimination.

Inspired by these developments, the proposed FSCA-EUNet introduces a Frequency–Spatial Cross-Attention (FSCA) module that jointly models frequency and spatial information to enhance texture sensitivity and global context awareness. The FSCA module effectively bridges the gap between spatial attention and frequency-domain representation, enabling more discriminative learning under visually repetitive conditions. Together with NonLocal blocks for long-range dependency modeling and MBConv for lightweight feature extraction, FSCA-EUNet achieves a balance between accuracy and efficiency, facilitating real-time deployment on edge devices.

While FSCA-EUNet effectively improves fine-grained classification performance and computational efficiency, it currently relies solely on RGB images, which may reduce robustness under varying illumination or complex industrial environments. Future work will explore the integration of multimodal data, such as RGB-D or hyperspectral imaging, to further enhance adaptability and generalization.

## 3. Materials and Methods

### 3.1. Methodology Overview

**Figure 1:**
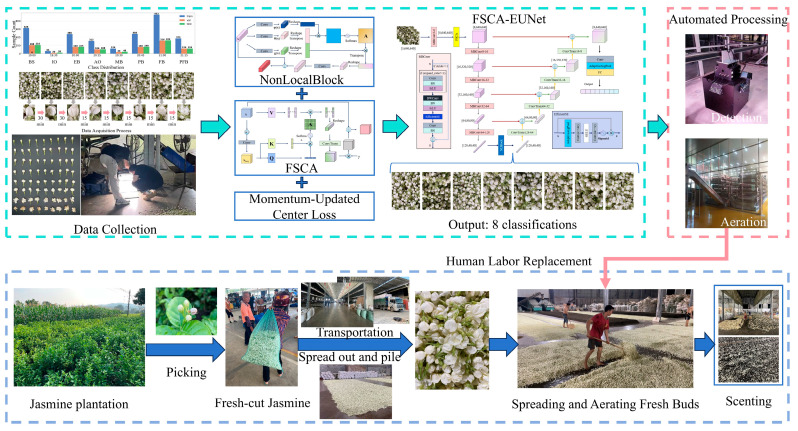
Schematic overview of the proposed research methodology.

Figure 1 illustrates the overall workflow of this study. After harvesting, transportation, postharvest spreading and aerating, jasmine flowers undergo the scenting process with tea leaves. During the aeration stage, images are collected for model training. These data are then processed by the proposed FSCA-EUNet framework, which integrates FSCA, NonLocalBlock, and momentum-updated center loss for fine-grained bloom stage classification. The classification results are finally applied to automated processing, replacing labor-intensive manual operations in tea scenting.

In the traditional jasmine tea scenting process, as illustrated in the second line of Figure 1, freshly harvested jasmine buds are manually collected, spread, and piled. During the subsequent flower-ventilation stage, the buds are aerated until blooming before being mixed with tea leaves for scenting. This stage relies heavily on manual monitoring, which is labor-intensive and subject to individual judgment. The proposed FSCA-EUNet replaces manual inspection in this stage with automated bloom-stage recognition, enabling objective and real-time monitoring of jasmine blooming dynamics. By integrating frequency–spatial cross-attention with intraclass consistency constraints, the model achieves accurate classification of subtle bloom differences, thereby enhancing both the efficiency and reliability of the scenting process.

### 3.2. Data Preparation

Image data of postharvest jasmine flowers during spreading and aeration were collected at a factory in Heng County, Guangxi, China, in September 2023. To capture the non-uniform temporal variation in bloom openness, which is characterized by slow initial changes, followed by accelerated progression, images of stacked flowers were acquired at 30 min intervals from 19:00 to 20:00 and then at 15 min intervals until 21:15, yielding 5728 images across 8 time intervals with a resolution of 1512 × 2016 pixels. The images were captured indoors under factory lighting, generally from a vertical viewing angle and at distances within approximately 1 m, ensuring clear visualization of stacked flowers while maintaining realistic operating conditions. Since the acquisition was conducted in factory settings, there was no strict requirement on the time of day, and variations in distance and illumination were naturally included to enrich the dataset and improve model robustness. The bloom-stage categories were defined according to the morphological features of jasmine petals, including the degree of opening, curvature, and overlap of petals, rather than acquisition time. The temporal intervals served only as a reference to ensure coverage of different morphological stages. All images were manually verified and labeled by experienced tea-processing technicians based on visual appearance, ensuring consistent and interpretable class definitions across the dataset.

**Figure 2:**
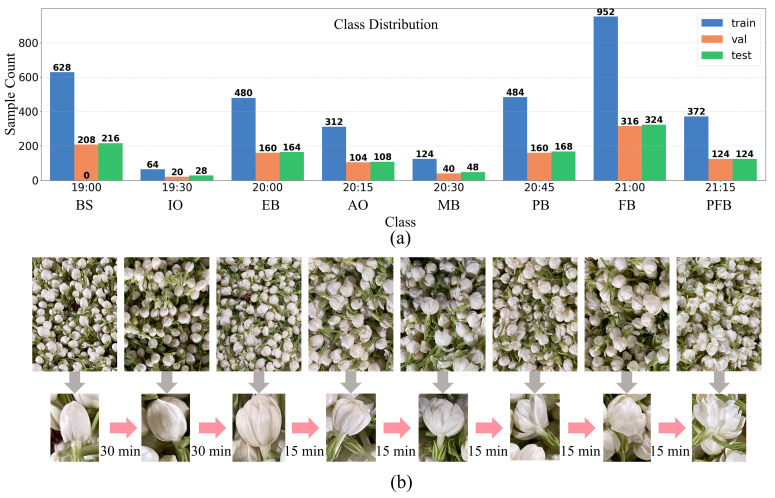
Class distribution and visual examples of jasmine bloom stages. (**a**) Class distribution of training, validation, and test datasets across eight bloom stages; (**b**) Sequential visual representation of jasmine flower opening over time intervals.

The dataset was divided into training, validation, and test sets at a 3:1:1 ratio, resulting in 3436 images for training, 1146 for validation, and 1146 for testing. Data augmentation techniques, including random resized crop, random horizontal flip, and normalization, were applied only to the training set in an online manner with a batch size of 16. In each epoch, the model was trained on all 3436 training images, but each batch of 16 images was randomly transformed, thereby enriching sample diversity without altering the overall dataset size. The temporal distribution and representative samples are shown in Figure 2a.

Representative image samples from all eight categories, illustrating the progressive bloom openness, are visually summarized in Figure 2b. The entire spreading and aeration process lasted approximately 2 h, during which a gradual increase in the number of postharvest jasmine flowers reaching bloom was observed. Notably, bloom progression accelerated significantly during the final 30 min due to cumulative heat generated by already opened flowers. This thermally driven self-reinforcing mechanism, which is strongly temperature-dependent, further promoted bud blooming. To intuitively reflect the phenological stage corresponding to each capture time, the eight time-point categories were renamed according to the typical bloom openness state: Bud Stage (BS, 19:00), Initial Opening (IO, 19:30), Early Bloom (EB, 20:00), Accelerated Opening (AO, 20:15), Mid Bloom (MB, 20:30), Peak Bloom (PB, 20:45), Full Bloom (FB, 21:00), and Post-Full Bloom (PFB, 21:15). This nomenclature captures the characteristic non-uniform temporal progression of jasmine flower opening, which exhibits slow initial changes followed by accelerated development, as evidenced by the shift to 15 min intervals after 20:00. The uneven sample distribution across categories, particularly the peak at Full Bloom, reflects the natural aggregation of flowers reaching this pivotal stage.

Interclass variations within the dataset were primarily manifested in two aspects: (1) gradational differences in bloom openness levels and (2) quantitative disparities in bloom density. Despite the subtle distinctions between classes, precise monitoring of bloom stages is critical for initiating scenting processes at peak fragrance intensity, which is essential for optimizing jasmine tea quality—the primary challenge addressed in this study.

### 3.3. Network Architecture

**Figure 3:**
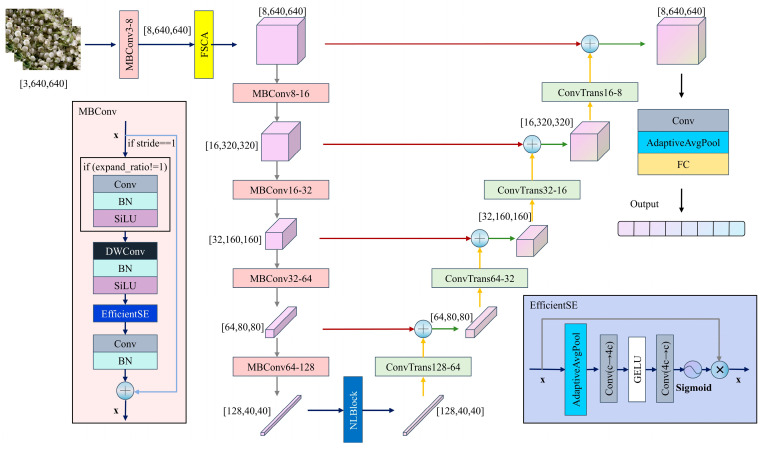
Frequency–Spatial Cross-Attention—Efficient U-shape Network (FSCA-EUNet).

As shown in Figure 3, the proposed FSCA-EUNet employs a U-Net encoder–decoder architecture, which is a neural network design that effectively captures both global context and local details of the input data. The encoder extracts hierarchical feature representations by progressively reducing spatial dimensions, while the decoder reconstructs detailed feature maps from these representations. Mathematically, given an input image **x**, the encoder produces feature maps **F** = Encoder(**x**), and the decoder reconstructs output features **Y** = Decoder(**F**). Skip connections between encoder and decoder layers help retain fine-grained information that may be lost during encoding. In fine-grained classification, subtle differences between classes often occur in small, localized regions. The encoder–decoder structure is particularly suitable for this task because the decoder can recover detailed spatial information while the encoder captures the overall context. This allows the network to focus on discriminative features across different regions, improving classification accuracy.

Building on this foundation, FSCA-EUNet enhances feature extraction for fine-grained recognition. The encoder hierarchically extracts multiscale features through five MBConv modules with depthwise separable convolutions to reduce computational complexity, whereas NonLocalBlock captures long-range dependencies in deeper layers. The decoder progressively recovers spatial resolution via transposed convolutions (ConvTranspose), integrates multiscale detail features through skip connections, and outputs classification results through adaptive pooling and fully connected layers. A FSCA mechanism is embedded after the initial encoder, and the center loss function constrains feature distributions to improve fine-grained classification performance.

MBConv employs an “expand-compress” inverted residual design. The channel dimensions are first expanded via a 1 × 1 convolution, followed by spatial feature extraction via a depthwise separable convolution, and finally compressed via a projection layer with a residual connection. The integrated EfficientSqueezeExcite module implements lightweight channel attention through global pooling and two-layer cascaded convolutions for dynamic feature recalibration, achieving 30% fewer parameters than standard SE modules while enhancing nonlinear representation via GELU activation.

### 3.4. Frequency–Spatial Cross-Attention Mechanism

**Figure 4:**
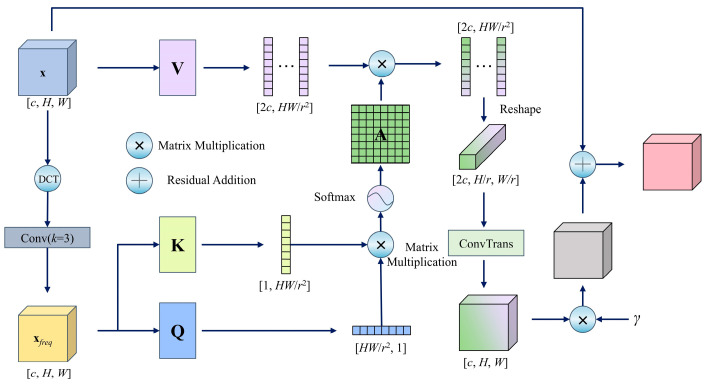
Frequency–Spatial Cross-Attention Mechanism (FSCA).

The proposed FSCA approach introduces a novel attention mechanism that integrates frequency-domain analysis with cross-scale interactions. Its core principle leverages frequency-domain features to guide spatial attention weight generation, thereby enhancing the joint modeling ability of the model for high-frequency textures and multiresolution features, as illustrated in Figure 4.

The module first applies a two-dimensional (2D) discrete cosine transform (DCT) to the input feature map **x**, mapping the spatial features to the frequency domain. A 3 × 3 convolutional layer subsequently extracts high-frequency components **x***_freq_*, which enhances the representation of edge and texture details. To reduce computational complexity, a cross-scale interaction strategy is adopted: the query (**Q**) and key (**K**) are downsampled to (*H*/*r*, *W*/*r*) resolution via 1 × 1 convolutions with stride *r*, reshaped into matrices **Q** and **K**, and used to compute the cross-position similarity scores. The scores are normalized by Softmax to generate the attention weight matrix **A**. The original input **x** is processed through an independent downsampled channel-expansion layer to produce the value vector **V**. After aggregating **V** with **A**, the output is upsampled via transposed convolution to restore the original resolution, residual-connected to the input features, and dynamically scaled by a learnable parameter *γ* to modulate the attention intensity. The final output is expressed in Equation (1),

(1) $$\mathrm{FSCA}\left(x\right)=\gamma \cdot \mathrm{DCT}{\left(\mathrm{Reshape}{\left({\left[V_{x}\right]}_{2c\times N}{\left[{\left({\left[Q_{x_{freq}}\right]}_{N\times 1}{\left[K_{x_{freq}}^{T}\right]}_{1\times N}\right)}^{T}\right]}_{N\times N}\right)}_{2c\times \frac{H}{r}\times \frac{W}{r}}\right)}_{c\times H\times W}+x_{c\times H\times W}$$

where **x** is the input feature map of size *c* (channel) × *H* (Height) × *W* (Width), DCT(·) denotes the 2D discrete cosine transform, Reshape(·) is a tensor reshaping operation to adjust the feature map dimensions for attention calculation, *r* is the downsampling rate, *N* = *HW*/*r*^2^ is the number of positions after downsampling, **x***_freq_* is the high-frequency component extracted from **x**, **Q_x_***_freq_*, **K_x_***_freq_*, and **V**_x_ are the query, key, and value matrices used in the attention computation, *γ* a learnable scalar controlling the attention intensity.

The FSCA module employs DCT to extract high-frequency features by decomposing images into cosine components of varying frequencies, linearly projecting spatial signals into the frequency domain to effectively separate low-frequency contours from high-frequency details (e.g., edges and textures). Its 2D form (DCT-2D), which is renowned for its energy compaction property, concentrates the majority of the information in the low-frequency region, as expressed in Equation (2),

(2) $$\begin{array}{l} \mathrm{DCT}\left(x\right)=\alpha \left(u\right)\alpha \left(v\right)\sum_{i=0}^{H-1}\sum_{j=0}^{W-1}x\left(i,j\right)\cos\left(\frac{\pi u\left(2i+1\right)}{2H}\right)\cos\left(\frac{\pi v\left(2j+1\right)}{2W}\right)\\ \alpha \left(k\right)=\left\{\begin{array}{ll} \sqrt{\frac{1}{N}}, & k=0\\ \sqrt{\frac{2}{N}}, & k\neq 0 \end{array}\right.\left(N=\begin{array}{ccc} H & or & W \end{array}\right) \end{array}$$

where **x**(*i*, *j*) denotes the spatial-domain input, (*u*, *v*) denote the frequency-domain outputs, and *H* and *W* are the height and width of the input feature map. *α*(*k*) is the normalization factor defined below, where N denotes the size of the signal along the dimension under consideration: *N* = *H* when computing along the vertical dimension and *N* = *W* when computing along the horizontal dimension.

The FSCA module explicitly extracts high-frequency texture features via DCT transformation, enabling cross-scale interaction with the original spatial features. This design leverages the energy concentration property of the frequency domain to enhance the model’s sensitivity to edges and local details, thereby addressing the limitations of traditional spatial attention in modeling high-frequency information. In addition, the model constructs a global attention matrix in downsampled space, restores resolution through transposed convolution, and adaptively balances dual-domain feature contributions via a dynamic weight modulation unit. This approach reconciles large receptive fields with low computational overhead, effectively resolving the conflict between detailed feature capture and computational efficiency in complex scenarios.

### 3.5. NonLocalBlock

**Figure 5:**
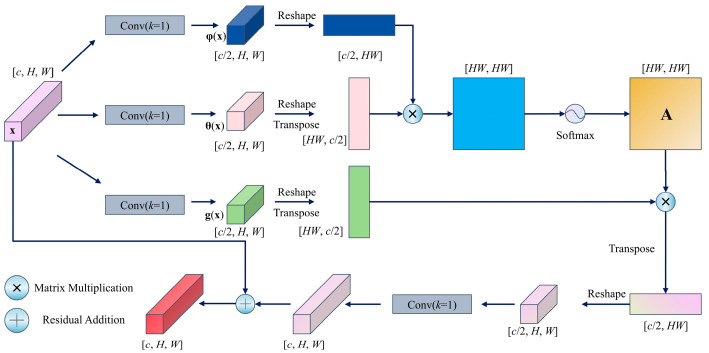
NonLocalBlock.

NonLocalBlock is a global context modeling module based on a self-attention mechanism. Its core principle establishes long-range dependencies between all spatial positions in feature maps, thereby enhancing the ability of the model to capture global information, as illustrated in Figure 5.

The module first processes the input feature map **x** through three 1 × 1 convolutions to generate three feature projections, namely, **θ**(**x**), **φ**(**x**), and **g**(**x**), with the channel dimensions compressed to half of the original input to reduce computational complexity. Subsequently, a spatial similarity matrix is computed via matrix multiplication between **θ**(x) and **φ**(**x**) and normalized into attention weights **A** via Softmax. The weighted aggregation of **A** and **g**(**x**) produces globally contextualized features, which are then adjusted to match the channel dimensions via a 1 × 1 convolution and combined with the original input **x** through a residual connection, as expressed in Equation (3),

(3) $$\mathrm{NL}\left(x\right)=\mathrm{Conv}{\left(\mathrm{Reshape}{\left({\left[\mathrm{softmax}\left(\varphi \left(x\right)\theta {\left(x\right)}^{T}\right)\right]}_{HW\times HW}{\left[g{\left(x\right)}^{T}\right]}_{HW\times \frac{c}{2}}\right)}_{\frac{c}{2}\times H\times W}\right)}_{c\times H\times W}+x_{c\times H\times W}$$

where x is the input feature map (*c* × *H* × *W*), Conv(·) denotes a 1 × 1 convolution restoring origional channel dimensions, Reshape(·) is a tensor reshaping operation to adjust the feature map dimensions for matrix operations, softmax(·) applies row-wise normalization to generate attention weights. **θ**(**x**), **φ**(**x**), and **g**(**x**) are channel-compressed projections computed via separate 1 × 1 convolutions at c/2 channels each. This nonlocal interaction mechanism addresses the limited receptive field of conventional convolution operations, particularly enhancing cross-regional feature correlations in complex recognition tasks. By strengthening robustness against occlusions and deformations, the nonlocal interaction mechanism significantly enhances global semantic comprehension.

### 3.6. Momentum-Updated Center Loss

During the training of FSCA-EUNet, the center loss is introduced as an auxiliary supervision signal and is jointly optimized with conventional cross-entropy loss to enhance intraclass compactness and interclass separability in the feature space. This loss function constrains the distance between sample feature vectors and their corresponding class centers, forcing samples of the same class to cluster in the embedding space, thereby alleviating classification ambiguity caused by excessive intraclass variations. Specifically, for the current training batch B with input features *f_i_* ∈ **R***^d^* and class labels *y_i_*, the center loss *L_C_* is given by Equation (4).

(4) $$L_{C}=\frac{\lambda }{2}\sum_{i=1}^{B}{\left\|f_{i}-c_{y_{i}}\right\|}_{2}^{2}+\eta {\left\|C\right\|}_{F}^{2}$$

where *c_yi_* ∈ **R***^d^* denotes the learnable center vector of class *y_i_*, **C** = [*c*_1_, …, *c_K_*]^⊤^ denotes the matrix composed of all class centers, and where *λ* and *η* control the loss intensity and regularization weight, respectively. During computation, the squared Euclidean distance between each feature vector *f_i_* and its corresponding class center *c_yi_* is calculated sequentially for B samples in the batch, and the loss values are summed over all samples. The squared Frobenius norm of the class center matrix **C** is applied to constrain the magnitude of all class center vectors, which prevents excessive deviation from the origin during training and model overfitting.

The class centers are dynamically adjusted via a momentum update strategy, as expressed in Equation (5).

(5) $$c_{y}^{\left(t\right)}=\left(1-\alpha \right)c_{y}^{\left(t-1\right)}+\alpha \cdot \frac{1}{\left|B_{y}\right|}\sum_{i\in B_{y}}f_{i}$$

where *c_y_*^(*t*)^ is the class center vector of class *y* at training epoch *t*, *α* denotes the momentum coefficient (default: 0.9), and **B***y* denotes the index set of samples belonging to class *y* in the current batch, *f_i_* is a feature vector extracted by the backbone network for the *i*-th sample. This strategy mitigates the drastic center oscillations caused by per-batch noise, progressively refining class center positions to capture the dynamic evolution of feature distributions during training. When jointly optimized with cross-entropy loss, the center loss forces features of the same class to cluster around their center (reducing intraclass variance), and the cross-entropy loss ensures separation between different class centers (increasing interclass distance). This synergistic optimization effectively minimizes feature intraclass variance in fine-grained classification tasks, improves classification performance, and enhances robustness in complex scenarios.

## 4. Results and Discussion

### 4.1. Experimental Results and Analysis

This study employed data augmentation techniques during training, including random resized crop, random horizontal flip, and normalization, to enhance model generalizability. The input images first underwent data preprocessing before being fed to the selected deep learning model for forward propagation. The cross-entropy loss was adopted as the primary loss function, and the Adam optimizer was employed for gradient updates with a learning rate of 0.001. For the FSCA-EUNet model, center loss was introduced to enhance interclass separability. The training was performed using minibatch gradient descent with a batch size of 16, and commonly adopted hyperparameter settings were applied to ensure stable convergence. All experiments were conducted on a workstation equipped with an Intel Core i9-13900K CPU, 64 GB RAM, and an NVIDIA RTX 4090 GPU (24 GB VRAM), running Ubuntu 22.04 and PyTorch 2.1.1, and Python 3.10.0. The same trained model was subsequently validated on a Jetson Orin NX (16 GB) to evaluate real-time inference performance and verify deployment consistency. The final performance was evaluated based on accuracy (A), precision (P), recall (R), and F1-score (F1) on the training and validation sets. These metrics are defined in Equation (6), where TP denotes true positives (predicted as positive and actually positive), FP denotes false positives (predicted as positive but actually negative), TN denotes true negatives (predicted as negative and actually negative), and FN denotes false negatives (predicted as negative but actually positive).

(6) $$\begin{array}{l} Accuracy=\frac{TP+TN}{TP+FP+FN+TN},\\ Precision=\frac{TP}{TP+FP},\\ Recall=\frac{TP}{TP+FN},\\ F_{1}=2\times \frac{Precision\times Recall}{Precision+Recall} \end{array}$$

The model was trained for 300 epochs, and the best-performing model was saved post-training to ensure generalizability on the test set. The classification results for each category on the training, validation, and test sets are presented in Table 1.

**Figure 6:**
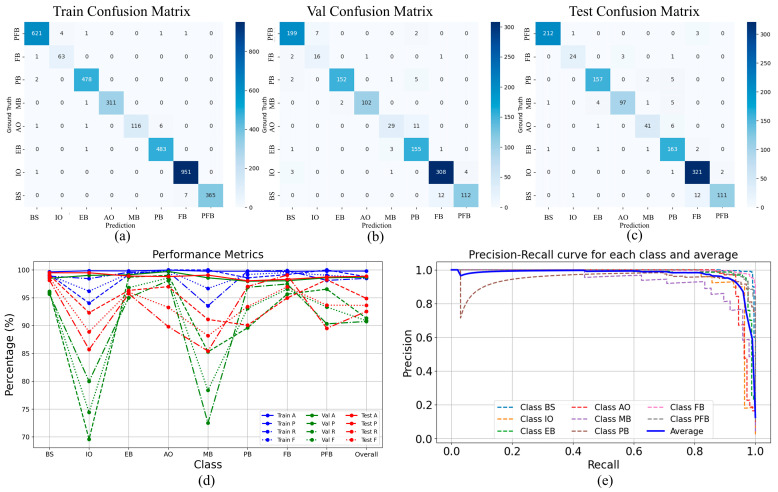
Confusion matrices and performance evaluation of the proposed FSCA-EUNet model across training, validation, and testing datasets. (**a**) Train Confusion Matrix; (**b**) Val Confusion Matrix; (**c**) Test Confusion Matrix; (**d**) Comparison of accuracy (A), precision (P), recall (R), and F1-score (F) for each class and overall across the three datasets; (**e**) PR curves for individual classes and the average performance.

The confusion matrices for the training, validation, and test sets are shown in Figure 6a–c, respectively. On the test set, the model achieved an overall precision of 95.52%, recall of 95.42%, and F1-score of 95.40%.

As shown in Table 1 and Figure 6d, the “IO” and “MB” categories exhibited relatively lower performance due to the limited sample quantities. However, their precision exceeded 90%, recall exceeded 85%, and F1-scores remained above 88% on the test set, demonstrating robust classification ability for small-sample categories. The precision-recall (PR) curve of the model is illustrated in Figure 6e, from which the mAP (Mean Average Precision) is computed, as defined in Equation (7),

(7) $$mAP=\frac{1}{C}\sum_{i=1}^{C}\underset{AP_{i}}{\underset{︸}{\sum_{n=1}^{N}\left(R_{n}^{i}-R_{n-1}^{i}\right)\cdot P_{n}^{i}}}$$

where mAP denotes the mean Average Precision, calculated as the average of the AP*_i_* values over all classes; AP*_i_* is the Average Precision for the *i*-th class, obtained as the area under the precision-recall (PR) curve for that class; *C* represents the number of classes; and *N* is the number of sampling points used to approximate the PR curve. The proposed method achieves an mAP of 97.24% on the test set.

### 4.2. Ablation Study

To validate the effectiveness of the proposed modules, ablation studies were conducted on individual components, including FSCA, NonLocalBlock, and center loss. The ablation experiment results are compared in Table 2.

**Figure 7:**
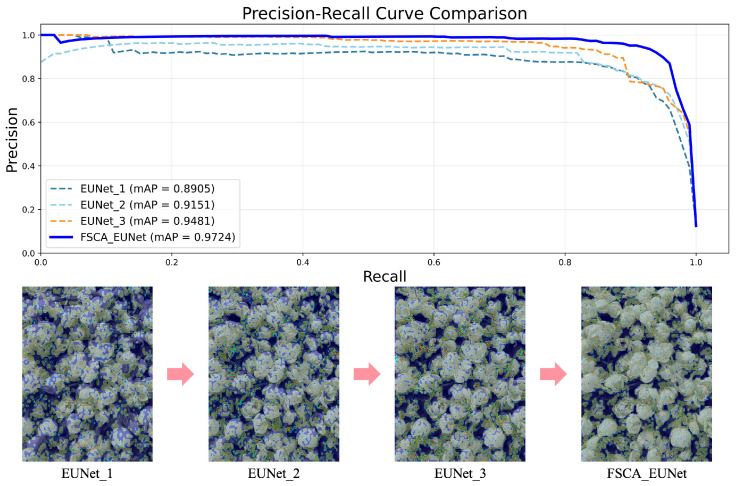
Ablation experiment results.

The ablation results demonstrate the progressive performance improvements contributed by each module. The baseline model achieves an mAP of 89.05%. Incorporating the FSCA module increases the mAP to 91.51%, with precision (P), recall (R), and F1-score exceeding 91%, validating the effectiveness of the proposed cross-attention mechanism. Adding the NonLocalBlock module further increases the P, R, and F1-score values to above 92.5%, and the mAP increases to 94.81%, demonstrating the importance of global context modeling. The final integration of the center loss achieves comprehensive optimization, with the P and R values exceeding 95% and the F1-score and mAP reaching 95.40% and 97.24%, respectively, highlighting the critical role of intraclass consistency constraints. The full model significantly outperforms the baseline model through component synergy, demonstrating the necessity of multimodule codesign. As shown in Figure 7, the PR curves of the ablation models confirm progressive performance gains, and the heatmaps reveal enhanced attention to the petal details, indicating continuous improvements in fine-grained discrimination performance.

### 4.3. Comparison with Other Attention Modules

To further evaluate the effect of the FSCA module, it was replaced with several representative attention mechanisms, including SE, CBAM, ECA, Coordinate Attention, and FcaNet, while keeping all other network structures identical. As shown in Table 3, the performance differences among the top-performing variants (FSCA-EUNet, FcaNet-EUNet, and SE-EUNet) are relatively small, with all achieving over 95% in precision, recall, and F1-score, and around 97% in mAP. FSCA-EUNet achieved the highest recall (95.42%) and F1-score (95.40%), indicating a stable balance between precision and recall, whereas FcaNet-EUNet and SE-EUNet slightly surpassed in precision and mAP. CBAM-EUNet and ECA-EUNet performed comparably but showed minor fluctuations across different metrics. The Coordinate Attention variant exhibited a more evident drop in performance, suggesting that coordinate encoding alone is insufficient for handling repetitive fine-grained patterns. Overall, all variants achieved comparable efficiency (15.4 G FLOPs, 0.88 M parameters, and 142 FPS), and the results confirm that the proposed Frequency–Spatial Cross-Attention (FSCA) provides competitive and balanced performance without additional computational cost.

### 4.4. Experimental Comparison with Different Classification Models

To ensure a fair comparison, all baseline models and the proposed FSCA-EUNet were trained under the same hyperparameter settings: 300 epochs, a batch size of 16, and an input resolution of 640 × 640. Data augmentation included random resized cropping and horizontal flipping, with normalization following ImageNet statistics. The Adam optimizer (lr = 0.001) and CrossEntropyLoss were adopted, while FSCA-EUNet additionally employed a momentum-updated center loss to improve class separability. These hyperparameters were selected based on practical considerations and common recommendations. The batch size, chosen as a power-of-two and limited by GPU memory, helps stabilize training and improve performance. The 300 epochs ensured full convergence, and the input resolution preserved fine image details for accurate feature extraction, while the optimizer and learning rate followed standard practice for similar tasks.

Comparative experiments were conducted between the proposed network and widely used models on the test set, as shown in Table 4. To better illustrate the improvement, ΔF1, defined as ΔF1 = F1_FSCA-EUNet_ − F1_baseline_, quantifies the relative enhancement in F1-score of FSCA-EUNet over each baseline model. The results demonstrate that the proposed FSCA-EUNet achieves superior performance across all key metrics, with accuracy of 92.55%, precision of 95.52%, recall of 95.42%, F1-score of 95.40%, and mAP of 97.24%. Although ResNet18 exhibits competitive accuracy (91.70%) and recall, it requires 11.18 M parameters—nearly 12 times larger than FSCA-EUNet (0.879 M). DenseNet121 shows a similar situation with over 6 M parameters but lower accuracy (86.81%). In contrast, lightweight models such as MobileNetV3-Small and EfficientNet-B0 provide high efficiency and faster inference speeds (e.g., 634.06 FPS and 288.11 FPS, respectively), but at the cost of reduced precision and recall. FSCA-EUNet achieves the best balance, delivering the highest overall accuracy while maintaining a drastically smaller parameter size and a reasonable inference speed (142.10 FPS), ensuring both effectiveness and suitability for real-time deployment on resource-constrained devices.

**Figure 8:**
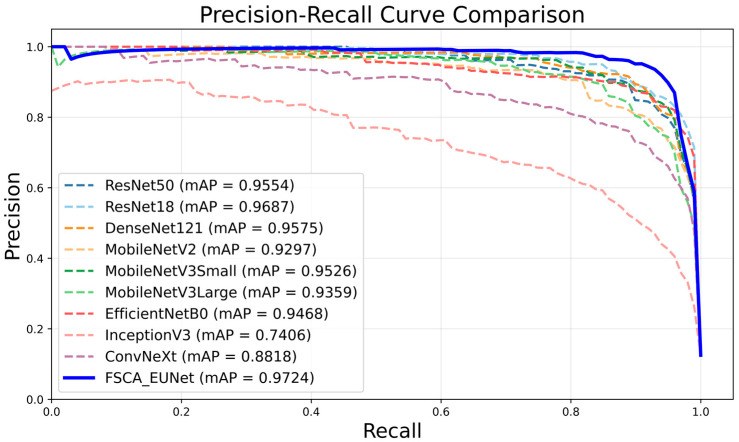
Comparison of PR curves.

The PR curves of all models are shown in Figure 8. Analysis based on these curves reveals that the proposed FSCA-EUNet significantly outperforms the comparison models, with its curve closest to the upper-right corner, demonstrating sustained high precision at increased recall rates. This validates the effectiveness of the proposed network in balancing detection sensitivity and false positive suppression, thereby establishing it as the optimal solution for the target task.

The superior performance of FSCA-EUNet over other algorithms mainly stems from its ability to address two key challenges in stacked jasmine flower images—highly repetitive backgrounds and very small inter-class differences between bloom stages. Most compared algorithms, such as conventional CNN-based classifiers and standard U-Net variants, operate solely in the spatial domain and thus have limited sensitivity to subtle texture differences. In contrast, FSCA-EUNet enhances fine-grained discrimination by fusing frequency-domain features (via DCT) with spatial cues in the FSCA module, enabling more accurate recognition of subtle textures than spatial-only models. Its context awareness is further improved through non-local blocks that capture long-range dependencies, suppress redundant background information, and highlight discriminative regions. The momentum-updated center loss increases class separability by enforcing intra-class compactness and inter-class distinction. Finally, the lightweight U-Net backbone preserves multi-scale features while maintaining low computational cost, reducing overfitting and improving generalization compared with heavier architectures.

### 4.5. Deployment with TensorRT

**Figure 9:**
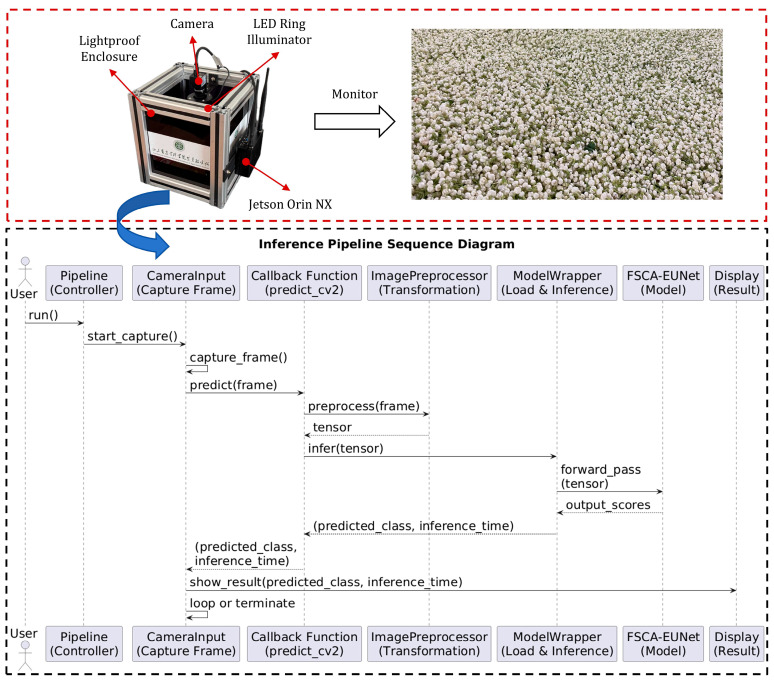
Jasmine Flower Bloom Stage Monitoring System.

To verify the practical deployment potential of FSCA-EUNet in industrial environments, a monitoring system was integrated into the production line. As shown in Figure 9, the system consists of a lightproof enclosure, an LED ring illuminator, an IRAYPLE MV-A3200CU000 camera (1920 × 1080 resolution), and an NVIDIA Jetson Orin NX.

A corresponding sequence diagram further details the system workflow. The process begins with the user initiating the pipeline via the run() method of the pipeline controller. Subsequently, the CameraInput module activates and continuously captures frames from the camera. For each captured frame, the predict_cv2() callback function is invoked to perform inference. This function first preprocesses the frame using the ImagePreprocessor to convert raw image data into a suitable tensor format. The preprocessed tensor is then forwarded to the ModelWrapper, which executes the forward pass through the underlying neural network model(FSCA-EUNet). The predicted class along with the inference time is returned back through the call stack to the CameraInput module. Finally, the prediction results are displayed to the user in real-time, with the cycle repeating for subsequent frames until termination. The predicted results are simultaneously transmitted to the central control system. Once the system determines that the bloom stage is complete, the central controller automatically initiates the next step of transporting jasmine flowers to mix with tea leaves for scenting.

To further verify deployment feasibility, the same trained FSCA-EUNet model was tested on an NVIDIA Jetson Orin NX (16 GB) using the PyTorch environment with FP32 precision. The inference results on the Jetson platform were consistent with those obtained on the workstation, confirming the reliability and portability of the proposed system for edge deployment.Experimental results showed that the pipeline achieved a throughput of 22.33 FPS with a latency of 44.78 ms per image, while maintaining 95.52% precision, 95.42% recall, 95.40% F1-score, and 97.24% mAP, consistent with offline experiments. These results demonstrate the model’s real-time deployment viability for in-factory jasmine bloom-stage monitoring, providing reliable visual feedback to support intelligent tea scenting automation.

### 4.6. Discussion

This study has proposed FSCA-EUNet, a lightweight model that integrates global feature modeling and intraclass consistency constraints, to recognize the bloom stages of stacked jasmine flowers in industrial settings. A high-resolution dataset capturing postharvest jasmine blooming processes was constructed through non-uniform interval data acquisition, providing foundational training data. The experimental results demonstrate that the model achieves 97.24% mAP on the test set, demonstrating significant improvement over baseline models, with only 29% parameters of ResNet18, thereby validating its efficiency and lightweight advantages in fine-grained classification. Comparative analysis reveals that FSCA-EUNet optimally balances recall and computational efficiency, maintaining F1-scores above 88% even in low-sample time intervals, offering reliable technical support for real-time scenting process monitoring.

The design of the Frequency–Spatial Cross-Attention (FSCA) module was motivated by the need to capture subtle inter-class differences in jasmine bloom stages while ensuring efficiency for edge deployment. Unlike self-attention or multi-head attention, which provide global modeling but incur high computational cost, FSCA integrates frequency-domain features via the discrete cosine transform to emphasize fine texture cues and suppress redundant background information. By applying cross-attention on downsampled tokens with lightweight projections, FSCA achieves a good balance between accuracy and efficiency, enabling FSCA-EUNet to remain compact while delivering superior classification performance.

To further assess the proposed FSCA module, it was compared with several representative attention mechanisms, including SE, CBAM, ECA, Coordinate Attention, and FcaNet, under identical network settings. As shown in Table 3, all variants achieved strong results, but FSCA-EUNet attained the best balance between precision and recall, with the highest recall (95.42%) and F1-score (95.40%), while maintaining comparable precision (95.52%) and mAP (97.24%). Despite similar FLOPs and parameters, FSCA-EUNet showed more stable and discriminative performance, confirming that the proposed Frequency–Spatial Cross-Attention effectively enhances fine-grained texture learning without extra computational cost.

Compared with previous studies, Sun et al. [18] employed RGB-D data of individual roses and showed that depth information consistently improved grading accuracy, with InceptionV3-RGBD achieving 98.44% validation accuracy. Fei et al. [19] further optimized efficiency by introducing a ShuffleNet-V2-based lightweight attention model (Opti-SA), which reached up to 99.915% accuracy on RGB-D datasets with only 0.38 M parameters and an inference time of 0.020 s. These results confirm the value of multimodal inputs and lightweight architectures, yet they were evaluated on isolated flowers rather than stacked samples. In contrast, Amer et al. [25] and Zhou et al. [26] focused on detection tasks: YOLOv5 and YOLOv7 were applied to jasmine flowers in natural field environments, reporting high precision and mAP@0.5 of 0.948 with real-time inference around 30 FPS. While these works highlight the advantages of RGB-D inputs or object detection frameworks, their problem settings differ from ours. Our study specifically targets fine-grained classification of stacked jasmine flowers in industrial production, where overlapping petals and highly repetitive backgrounds pose greater challenges. Under these conditions, the proposed FSCA-EUNet achieved 97.24% mAP and 22.33 FPS on Jetson Orin NX using only RGB images, competitive accuracy relative to multimodal methods, and practical deployability for real-time monitoring.

This study also has certain limitations. The dataset exhibits temporal imbalance, with fewer samples in the early blooming stages, which may affect model generalization under underrepresented conditions. The classification scheme was designed based on the morphological characteristics of stacked jasmine flowers rather than fixed temporal intervals, reflecting the overall blooming progression during the scenting process. This approach provides an initial and practical differentiation that aligns with industrial evaluation standards but inevitably introduces some subjectivity, as petals often overlap and transitions between adjacent stages are subtle. Moreover, adjacent bloom stages often show only subtle visual differences, and although the incorporation of Center Loss reduces intraclass variations, misclassification can still occur when stage boundaries are highly ambiguous. Another limitation is that the current framework relies solely on RGB imaging, which may reduce robustness under varying illumination or more complex environments.

Future research will focus on expanding multitemporal and balanced datasets across different production cycles, employing advanced augmentation and temporal modeling to better capture blooming dynamics, and exploring multimodal imaging modalities such as RGB-D or hyperspectral data to enhance discriminative capability. In addition, the refinement of the classification scheme through quantitative morphological descriptors (e.g., petal openness ratio, curvature index, and texture features) will be pursued to reduce subjectivity and improve reproducibility. In addition, hyperparameter optimization remains a key factor influencing model generalization and stability. Although the present work employed empirically tuned parameters to ensure convergence and fair comparison, future studies will explore systematic optimization strategies such as grid search or Bayesian optimization to further refine model performance.

FSCA-EUNet demonstrates the effectiveness of integrating frequency-domain features with spatial cross-attention for fine-grained image classification, providing new insights into lightweight attention mechanisms that balance global context modeling and computational efficiency. Practically, the model offers a robust and efficient tool for real-time monitoring of postharvest jasmine blooming, enabling automated quality assessment and process optimization in industrial settings. This approach can be extended to other agricultural and horticultural applications where subtle visual differences must be captured with limited computational resources, bridging the gap between advanced image analysis techniques and real-world deployment.

## 5. Conclusions

This study proposes FSCA-EUNet, a fine-grained classification model for recognizing the bloom stages of postharvest jasmine flowers under industrial stacking conditions. Built upon the efficient U-Net architecture, the proposed model integrates FSCA, NonLocalBlock, and momentum-optimized Center Loss to address the challenges of dynamic nonlinear blooming processes and subtle interclass variations. The experimental results demonstrate that the proposed model achieves 95.52% precision, 95.42% recall, 95.40% F1-score, and 97.24% mAP on the test set. The ablation studies revealed an 8.19% mAP improvement compared with the baseline EUNet model, thereby validating the efficacy of modular codesign. Compared to mainstream models, FSCA-EUNet maintains lower computational costs (15.445 G FLOPs) and outperforms ResNet18 by 1.81% and 3.72% in terms of precision and recall, respectively, with only 7.6% of its parameters (878.851 K). Notably, the proposed model maintains F1-scores above 88% in low-sample intervals (“IO, 19:30,” “MB, 20:30”) and exhibits superior PR curve characteristics, indicating balanced sensitivity and false positive suppression. This study provides quantitative decision-making support for automated jasmine tea scenting processes. Future research will expand multitemporal datasets and explore multimodal data fusion to further improve model performance.

## Figures and Tables

**Table 1:** Classification results.

Class	Train			Val			Test		
	P (%)	R (%)	F1 (%)	P (%)	R (%)	F1 (%)	P (%)	R (%)	F1 (%)
BS	99.20	98.89	99.04	96.14	95.67	95.90	99.07	98.15	98.60
IO	94.03	98.44	96.18	69.57	80.00	74.42	92.31	85.71	88.89
EB	99.17	99.58	99.38	98.70	95.00	96.82	96.32	95.73	96.02
AO	100.00	99.68	99.84	99.03	98.08	98.55	97.00	89.81	93.27
MB	100.00	93.55	96.67	85.29	72.50	78.38	91.11	85.42	88.17
PB	98.57	99.79	99.18	89.60	96.88	93.09	90.06	97.02	93.41
FB	99.17	99.89	99.53	95.65	97.47	96.55	94.97	99.07	96.98
PFB	100.00	98.12	99.05	96.55	90.32	93.33	98.23	89.52	93.67
Overall	99.19	99.18	99.18	94.90	94.79	94.78	95.52	95.42	95.40

**Table 2:** Comparison of ablation experiment results.

Model	FSCA	NonLocalBlock	Center Loss	P (%)	R (%)	F1 (%)	mAP (%)
EUNet_1	×	×	×	91.10	90.34	90.30	89.05
EUNet_2	√	×	×	91.39	91.78	91.10	91.51
EUNet_3	√	√	×	92.98	92.63	92.69	94.81
EUNet_4	√	√	√	95.52	95.42	95.40	97.24

**Table 3:** Comparison of Attention Mechanisms.

Model	A (%)	P (%)	R (%)	F1 (%)	mAP (%)	FLOPs	Param	FPS
SE-EUNet	93.98	95.27	95.17	95.19	97.29	15.438 G	878.851 K	142.80
CBAM-EUNet	94.00	94.68	94.24	94.33	96.67	15.438 G	878.851 K	143.72
ECA-EUNet	91.21	94.60	94.49	94.49	96.79	15.438 G	878.851 K	143.53
CoordAtt-EUNet	85.01	92.42	92.37	92.17	93.49	15.438 G	878.851 K	142.95
FcaNet-EUNet	93.02	95.53	95.25	95.30	97.29	15.438 G	878.851 K	143.30
FSCA-EUNet	92.55	95.52	95.42	95.40	97.24	15.445 G	878.851 K	142.10

**Table 4:** Comparison between different classification models.

Model	A (%)	P (%)	R (%)	F1 (%)	ΔF1 (%)	mAP (%)	FLOPs	Param	FPS
ResNet50	87.83	92.66	92.54	92.45	+2.95	95.54	31.823 G	23.524 M	92.35
ResNet18	91.70	94.69	94.66	94.59	+0.81	96.87	14.886 G	11.181 M	243.64
DenseNet121	86.81	92.74	92.46	92.24	+3.16	95.75	23.639 G	6.960 M	100.96
MobileNetV2	81.85	91.04	90.42	90.09	+5.31	92.97	1.104 G	71.064 K	404.66
MobileNetV3Small	86.69	93.45	93.39	93.22	+2.18	95.26	809.284 M	83.404 K	634.06
MobileNetV3Large	76.39	88.13	86.44	84.95	+10.45	93.59	1.076 G	45.304 K	814.70
EfficientNetB0	89.06	92.8	92.46	92.54	+2.86	94.68	1.443 G	55.768 K	288.11
InceptionV3	63.37	73.05	75.25	73.58	+21.82	74.06	3.858 G	69.568 K	759.84
ConvNeXt	75.89	87.97	88.14	87.1	+8.3	88.18	34.080 G	6.908 M	18.18
FSCA-EUNet(Ours)	92.55	95.52	95.42	95.40	-	97.24	15.445 G	878.851 K	142.10

## Data Availability

The data are not publicly available due to privacy and institutional policy restrictions but are available from the corresponding author upon reasonable request.
